# A prospective study examining cachexia predictors in patients with incurable cancer

**DOI:** 10.1186/s12904-019-0429-2

**Published:** 2019-06-04

**Authors:** Ola Magne Vagnildhaug, Cinzia Brunelli, Marianne J. Hjermstad, Florian Strasser, Vickie Baracos, Andrew Wilcock, Maria Nabal, Stein Kaasa, Barry Laird, Tora S. Solheim

**Affiliations:** 1 Department of Clinical and Molecular Medicine, Faculty of Medicine and Health Sciences, NTNU - Norwegian University of Science and Technology, Postbox 8905 MTFS, NO-7491 Trondheim, Norway; 2 Palliative Care, Pain Therapy and Rehabilitation Unit, Fondazione IRCCS Istituto Nazionale dei Tumori, via Giacomo Venezian 1, 20133 Milan, Italy; 3 European Palliative Care Research Centre (PRC), Department of Oncology, Oslo University Hospital, and Institute of Clinical Medicine, University of Oslo, Box 4956, Nydalen, 0424 Oslo, Norway; 4 Department of Internal Medicine and Palliative Care Centre, Cantonal Hospital, Oncological Palliative Medicine, Section Oncology, Rorschacher Strasse 95, CH-9007 St. Gallen, Switzerland; 5 Division of Palliative Care Medicine, Department of Oncology, University of Alberta, Cross Cancer Institute 11560 University Avenue, Edmonton, Alberta T6G 1Z2 Canada; 6 Nottingham University Hospitals NHS Trust, Hucknall Road, Nottingham, NG5 1PB UK; 7 Hospital Universitari Arnau de Vilanova and Universidad de Lleida, Av. Alcalde Rovira Roure 80, 25198 Lleida, Spain; 8 Edinburgh Cancer Research UK Centre, University of Edinburgh, Western General Hospital, Crewe Road South, Edinburgh, EH4 2XR UK; 9 Cancer Clinic, St. Olav’s Hospital, Trondheim University Hospital, Postboks 3250 Sluppen, NO-7006 Trondheim, Norway

**Keywords:** Cachexia, Pre-cachexia, Weight loss, Cancer, Palliative care

## Abstract

**Background:**

Early intervention against cachexia necessitates a predictive model. The aims of this study were to identify predictors of cachexia development and to create and evaluate accuracy of a predictive model based on these predictors.

**Methods:**

A secondary analysis of a prospective, observational, multicentre study was conducted. Patients, who attended a palliative care programme, had incurable cancer and did not have cachexia at baseline, were amenable to the analysis. Cachexia was defined as weight loss (WL) > 5% (6 months) or WL > 2% and body mass index< 20 kg/m^2^. Clinical and demographic markers were evaluated as possible predictors with Cox analysis. A classification and regression tree analysis was used to create a model based on optimal combinations and cut-offs of significant predictors for cachexia development, and accuracy was evaluated with a calibration plot, Harrell’s c-statistic and receiver operating characteristic curve analysis.

**Results:**

Six-hundred-twenty-eight patients were included in the analysis. Median age was 65 years (IQR 17), 359(57%) were female and median Karnofsky performance status was 70(IQR 10). Median follow-up was 109 days (IQR 108), and 159 (25%) patients developed cachexia. Initial WL, cancer type, appetite and chronic obstructive pulmonary disease were significant predictors (*p* ≤ 0.04). A five-level model was created with each level carrying an increasing risk of cachexia development. For Risk-level 1-patients (WL < 3%, breast or hematologic cancer and no or little appetite loss), median time to cachexia development was not reached, while Risk-level 5-patients (WL 3–5%) had a median time to cachexia development of 51 days. Accuracy of cachexia predictions at 3 months was 76%.

**Conclusion:**

Important predictors of cachexia have been identified and used to construct a predictive model of cancer cachexia.

**Trial registration:**

ClinicalTrials.gov Identifier: NCT01362816.

## Background

Cachexia is present in up to half of patients with cancer [1]. It adversely affects the well-being of many patients with cancer by inducing progressive weight loss as well as impairing appetite, physical function and quality of life [2]. Moreover, cachexia increases mortality and impedes delivery of anti-cancer treatment [3].

To date, there is no licensed treatment and no standard of care for patients with cancer cachexia. Corticosteroids have been shown to improve fatigue [4] and progestins have improved weight loss, however lack of positive effects on lean body mass, physical function or nutritional intake means that these agents have limited clinical benefits [5]. Further, the side effects of these drugs often outweigh potential benefits. Recently, selective androgen receptor modulators and ghrelin agonists have been examined in this area, however lack of demonstrable effects on lean body mass and/or function mean that these have not been granted regulatory approval for the treatment of cachexia [6, 7].

One of the reasons that the aforementioned and other agents may have proven inefficacious is that they may not have been used at the optimal time point and/or in patients truly at risk of developing cachexia. It has been argued that to optimise efficacy of cachexia medications, treatment should be initiated as early as possible [8]; even before cachexia is established, termed pre-cachexia. Pre-cachexia is the first part of a cachexia staging system based on a trajectory format with the latter two being cachexia and refractory cachexia [9]. In this staging system, cachexia was defined as more than 5% weight loss in 6 months, or more than 2% weight loss if low body mass index (< 20 kg/m^2^) or sarcopenia were present. Refractory cachexia ensues when the cancer becomes pro-catabolic and unresponsive to anti-cancer treatment.

Pre-cachexia was proposed as a stage where early clinical and metabolic signs such as anorexia and inflammation were present, but substantial weight loss was not [9]. The intention was to separate patients likely to develop cachexia from those who are not. However, diagnostic criteria were not suggested, and the challenge remains to optimally stratify patients into high and low risk groups. Several attempts at defining criteria for pre-cachexia have been made. These attempts have mostly been based on cross-sectional data or analyses of overall survival, study designs that are inadequate in showing if the criteria in question imply a greater risk of developing cachexia over time [10–12].

Therefore, the primary aim of this study was to identify which factors most strongly predict development of cachexia in a prospective cohort of patients with incurable cancer. Secondary aims were to construct a model to predict cachexia based on the optimal combinations and cut-offs of the identified predictors, and to evaluate the model’s accuracy.

## Methods

### Patients and study design

Between April 2011 and October 2013, 1739 patients from 30 centres across Europe (27), Canada (2) and Australia (1) were included in the European Palliative Care Cancer Symptom study (EPCCS). The participating centres are presented in the main publication originating from this study [13]. EPCCS was a prospective observational study conducted by the European Palliative Care Research Centre (PRC) (https://oslo-universitetssykehus.no/avdelinger/kreftklinikken/avdeling-for-kreftbehandling/prc) and the European Association for Palliative Care (EAPC) Research Network (https://www.eapcnet.eu/research/research-network). The overall aim of the EPCCS study was to improve the understanding of symptom development, and how these symptoms may best be assessed and classified in order to improve symptom management. Eligible patients met the following key inclusion criteria: ≥18 years of age; with incurable cancer and attending a palliative care program. Data pertaining to cancer cachexia were retrieved from this dataset and assessed as part of the present study.

### Data collection and assessments

Patients were assessed at baseline and then approximately every 4 weeks for at least three follow-up visits or until death. The following information was collected: Demographical data (age, gender, geographical region and treatment setting [inpatient, outpatient, home care]), disease specific data (cancer type and stage [localized vs. metastatic]), height, current body weight and patient reported weight loss in the 6 months prior to inclusion. Weight loss at subsequent visits was calculated by adding measured weight change to baseline reported weight loss. Cachexia was diagnosed at first occurrence of either a) weight loss > 5% since 6 months prior to inclusion or b) weight loss > 2% since 6 months prior to inclusion if current body mass index (BMI) < 20 kg/m^2^. All patients were assessed for cachexia at baseline. If anyone had insufficient data to be assessed for cachexia at baseline, data from the first follow up visit was used as baseline registrations if available. Only patients without cachexia at baseline were included in the analysis.

Performance status was assessed according to Karnofsky Performance Status (KPS) (0–100). Comorbidities in terms of heart disease, renal disease, chronic obstructive pulmonary disease (COPD) or arthritis were registered.

The dataset was not sufficiently large to assess the risk of cachexia development of each individual cancer type. Thus, cancer type was grouped a priori into one of three categories: Low risk - Breast cancer and haematological cancers (lymphoma, leukemia and myelomatosis); High risk – pancreatic and gastric cancer; Neutral risk - all other cancers. This was based on previous literature on cancer type and association with cachexia [14, 15].

The following patient reported outcome measurements (PROMs) were registered: Food intake was assessed as “less than usual”, “more than usual” or “unchanged” according to the abridged Patient-Generated Subjective Global Assessment (aPG-SGA) [16]. Physical and emotional functioning (0 [worst] - 100 [best]), anorexia and fatigue (0[best] – 100 [worst]) were assessed according to the European Organisation for Research and Treatment of Cancer Quality of Life Questionnaire (EORTC-QLQ) C15 PAL [17]. Both instruments are widely used, and well validated in the target population.

### Statistical considerations

Patients who developed cachexia during follow up were identified and the remaining patients were censored at the time of their last body weight registration. Time to cachexia development or censoring was calculated. Univariable Cox Proportional Hazards method was used to estimate hazard rate ratios (HR) for cachexia development with the following predictors assessed at baseline: Age, gender, cancer type (low risk, neutral risk or high risk), cancer stage, comorbidity, weight loss, BMI, performance status, physical functioning, emotional functioning, fatigue, food intake and appetite. All predictors with *p*-value < 0.20 were included in a multivariable Cox model. Multicollinearity among the candidate predictors was checked by estimating Spearman’s correlation coefficients. The least significant predictors were dropped from the multivariable model one by one in a manual backwards selection, until only significant predictors remained. All possible interactions among the remaining predictors were examined and added to the model if significant. Due to known association between COPD and lung cancer [18], a sensitivity analysis specifically adjusting for lung cancer alongside the a priori cancer type categorization (low risk, neutral risk, high risk) was performed.

In order to construct a model that uses optimal combinations and cut--offs of the identified risk factors to predict cachexia development, a classification and regression tree (CART) analysis for failure time data was used. CART is a non-parametric data-mining procedure which examines all possible cut-offs of every variable to create separate subgroups of significantly different risks. It repeatedly splits the population based on the variable and cut-off that most optimally stratifies risk of cachexia development in the current group. It stops when significant divisions no longer can be performed. The final subgroups were compared, and adjacent subgroups with similar risk of cachexia development were merged to create levels of increasing risk of cachexia development. Kaplan-Meier curves were plotted for each risk-level, and both log-rank test for differences in cachexia development probabilities among risk levels, and test for trend in cachexia development probabilities (i.e. cachexia development probability of Risk-level 1 ≤ Risk-level 2 ≤ … ≤ Risk-level 5) were performed. To assess accuracy of this model, a calibration plot was created by plotting predicted vs. observed risk at 3 months, and Harrell’s c-statistic was estimated to assess discriminatory capacity. In addition, receiver operating characteristic (ROC) curve analysis at 3 months in patients alive and still on study was performed. For the different possible cut-offs of a diagnostic test (all possible cut-offs for the “prediction function” from the model in this case), the ROC curve is a plot of the true positive rate (sensitivity, i.e. the proportion of patients correctly classified by the model among those actually developing cachexia) against the false positive rate (1- specificity, i.e. the proportion of patients wrongly classified as “developing cachexia” by the model, among those actually not developing cachexia). This was done to evaluate if a cut-off with both high sensitivity and high specificity for the prediction of cachexia development could be identified. A two-sided *p*-value of < 0.05 was considered significant in all analyses, unless stated otherwise. Stata version 13.1 (College Station, Texas, USA) was used for statistical analyses, and the Stata-module Cart [19] was used for the CART-analysis.

## Results

**Fig. 1:**
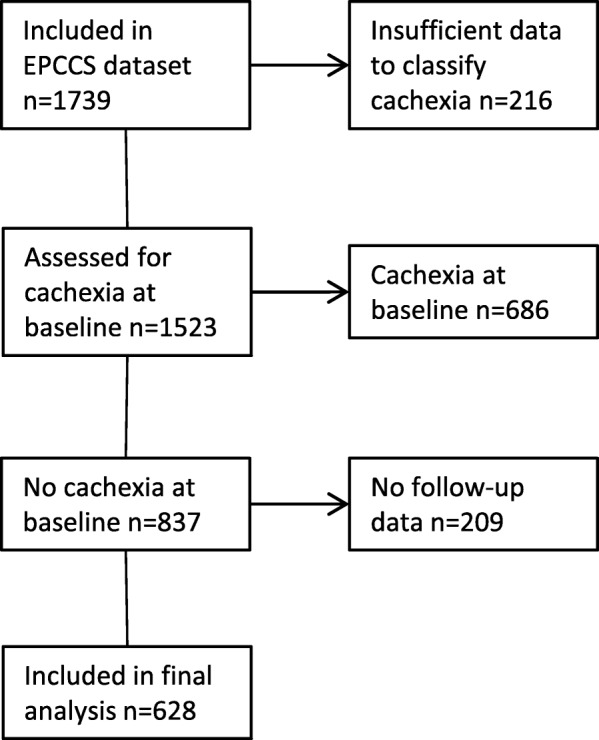
Flow chart

A flow chart of patient selection is shown in Fig. 1. Of the 1739 patients included in the EPCCS-study, 425 patients (24%) were excluded because of missing data necessary to classify patients as cachexic or not cachexic. Six-hundred-and-eighty-six patients (39%) already had cachexia at baseline and therefore were inadmissible to further analysis, leaving 628 (36%) patients to the final analysis.

Patient characteristics are shown in Table 1. The median age was 65 years (IQR 17), 359 (57%) were female and median KPS was 70 (IQR 10). One-hundred-and-fifty-nine patients (25%) developed cachexia during follow-up. For affected individuals, cachexia occurred in a median time of 63 days (IQR 79). Overall, minimum follow-up was 16 days and median follow-up was 109 days (IQR 108).

**Table 1:** Baseline patient characteristics

Patient characteristics (*n* = 628)		
Median Age at inclusion (IQR)	65	(17)
Gender f (%)		
Female	359	(57)
Male	269	(43)
Geographical region f (%)		
Europe	578	(92)
Canada	43	(7)
Australia	7	(1)
Cancer type f (%)		
Low risk cancer	184	(29)
Breast	171	(27)
Haematological	13	(2)
Neutral risk cancer	410	(65)
Lung	125	(20)
Colorectal	70	(11)
Prostate	48	(8)
Female genitalia	36	(6)
Head and neck	24	(5)
Urinary	20	(3)
Hepatobiliary	17	(3)
Sarcoma, connective and soft tissue	17	(3)
Small intestine	11	(2)
Oesophageal	8	(1)
Other	34	(5)
High risk cancer	33	(5)
Pancreatic	24	(4)
Gastric	9	(1)
Cancer stage f (%)		
Local	83	(13)
Metastatic/disseminated	543	(87)
Treatment setting		
Inpatients	56	(9)
Outpatients	483	(78)
Home care	77	(13)
Anti-cancer treatment		
Chemotherapy	337	(54)
Hormonal therapy	81	(13)
Radiotherapy	35	(6)
Other	47	(7)
No treatment	173	(28)
Median Karnofsky PS (IQR)	70	(10)
Weight loss (6 months) f (%)		
< 1%	535	(85)
1–5%	93	(15)
Mean BMI (SD)	25.5	(4.5)
Comorbidities f (%)		
Heart disease	165	(26)
COPD	62	(10)
Arthritis	51	(8)
Renal disease	17	(3)

Abbreviations: *IQR* interquartile range, *PS* performance status, *SD* standard deviation, *COPD* chronic obstructive pulmonary disease

Table 2 shows a univariable analysis of potential predictors of cachexia development. Gender, weight loss, performance status, food intake, appetite loss, cancer type and COPD were all significant predictors (*p* ≤ 0.02). In addition, physical functioning and cancer stage were included in the multivariable analysis due to *p*-values < 0.20. Physical functioning and KPS (0.53, *p* <  0.001) and appetite and food intake (0.54, p <  0.001) had correlation coefficients > 0.5. After manual backward selection, where insignificant predictors were removed one by one, weight loss, cancer type, appetite and COPD all remained significant (*p* ≤ 0.04) and were kept in the model (Table 3). A significant interaction (*p* <  0.01) was demonstrated between weight loss and cancer type, signifying that the effect of cancer type on risk of developing cachexia became less important if weight loss already was high. Thirty percent of patients with lung cancer had COPD in contrast to the overall COPD prevalence of 10%. Thus, a sensitivity analysis was performed adjusting for lung cancer alongside the a priori cancer type classification. This analysis showed that patients with lung cancer had a slightly higher risk of cachexia development compared to patients classified as having neutral risk cancer (HR [95%CI] 2.7 [1.4–5.2] vs 2.5 [1.4–4.3], respectively) and risk attributable to COPD fell slightly and COPD no longer significantly predicted cachexia development (HR 1.5 [0.9–2.6]).

**Table 2:** Univariable analysis

Univariable analysis			
	HR	95% CI	*p*
Age at inclusion	1.01	1.00–1.03	0.06
Gender			
Male	1.5	1.1–2.0	0.01
Female	1		
Weight loss (6 months)	1.6	1.4–1.7	< 0.001
BMI	0.99	0.95–1.03	0.53
Karnofsky PS (0–100)	0.98	0.97–1.00	< 0.01
Fatigue (0–100)	1.00	1.00–1.01	0.26
Physical functioning (0–100)	0.99	0.99–1.00	0.09
Emotional functioning (0–100)	1.00	0.99–1.01	0.67
Food intake			
Less than usual	1.7	1.2–2.4	< 0.01
More than usual	1.0	0.6–1.7	0.93
Unchanged	1		
Appetite loss (0–100)	1.01	1.01–1.02	< 0.001
Cancer stage			
Metastatic/disseminated	1.7	1.0–2.9	0.07
Local	1		
Cancer type^a^			
High risk cancer	4.4	2.4–8.1	< 0.001
Neutral risk cancer	2.1	1.4–3.1	< 0.001
Low risk cancer	1		
Heart disease	0.9	0.6–1.3	0.55
Renal disease	1.4	0.6–3.1	0.45
Arthritis	1.4	0.8–2.3	0.22
COPD	1.7	1.1–2.7	0.02

Abbreviations: *HR* hazard ratio, *CI* confidence interval, *BMI* body mass index, *PS* performance status, *COPD* chronic obstructive pulmonary disease

^a^Low risk - Breast cancer, lymphoma, leukaemia; High risk – pancreatic and gastric cancer; Neutral risk - all other cancers

**Table 3:** Multivariable analysis

Multivariable analysis			
	HR	95% CI	p
Weight loss	1.9	1.5–2.2	< 0.001
Cancer type^a^			
Low risk	1		
Neutral risk	2.5	1.5–4.3	< 0.01
High risk	6.3	2.9–13.8	< 0.001
Appetite loss (0–100)	1.005	1.000–1.011	0.04
COPD	1.6	1.0–2.6	0.04
Interactions with weight loss			
Medium risk cancer	0.8	0.7–1.0	0.06
High risk cancer	0.6	0.4–0.9	< 0.01

Abbreviations: *HR* hazard ratio, *CI* confidence interval, *COPD* chronic obstructive pulmonary disease

^a^Low risk - Breast cancer, lymphoma, leukaemia; High risk – pancreatic and gastric cancer; Neutral risk - all other cancers

**Fig. 2:**
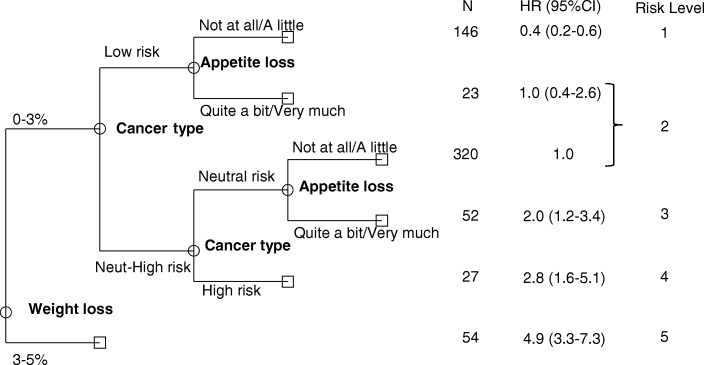
Classification and regression tree (CART) analysis. The study population is divided repeatedly according to optimal cut-offs of the variables weight loss (rounded to the nearest integer), cancer type and appetite loss into subdivisions of significantly different hazard rates. Adjacent subdivisions from different branches with similar hazard rates are combined resulting in five risk-levels. Hazard ratios (HR) are reported relative to the branch with neutral risk cancer type and no or little appetite loss

Figure 2 shows the CART-analysis. Weight loss, cancer type and appetite loss could be used to identify six groups of patients, each with a homogenous risk of cachexia development within the group. Two groups from adjacent branches of the classification and regression tree were combined due to similar hazard ratios, resulting in a model of five levels of increasing risk of cachexia development:

1. < 3% weight loss, low risk cancer type and no/little appetite loss
2. < 3% weight loss and either low risk cancer type and quite a bit/very much appetite loss OR neutral risk cancer type and no/little appetite loss
3. < 3% weight loss, neutral risk cancer type and quite a bit/very much appetite loss
4. High risk cancer type
5. 3–5% weight loss

**Fig. 3:**
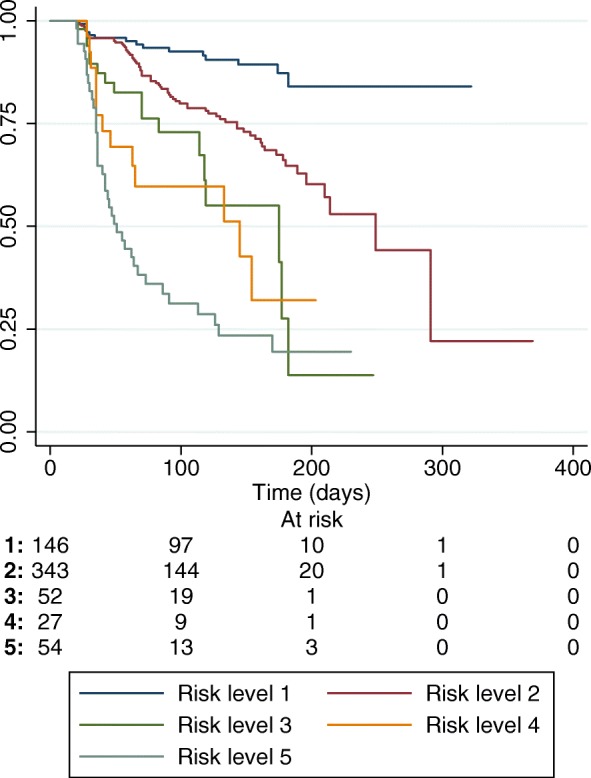
Kaplan-Meier plot of time to cachexia development depending on risk-level. Median time to cachexia development was not reached in level 1, 249 days for level 2, 175 days for level 3, 145 days for level 4 and 51 days for level 5. Log-rank test and test for trend in failure time-analysis were both significant (*p* <  0.0001)

Figure 3 shows the Kaplan-Meier curves for time to cachexia development in these five risk-levels. Median time to cachexia development was not reached in Risk-level 1, 249 days for Risk-level 2, 175 days for Risk-level 3, 145 days for Risk-level 4 and 51 days for Risk-level 5. Log-rank test for differences in cachexia development probabilities and test for trend in cachexia development probabilities were both significant (*p* <  0.0001), confirming that probability of cachexia development not only differed between levels, but was increasing with increasing risk-level.

**Fig. 4:**
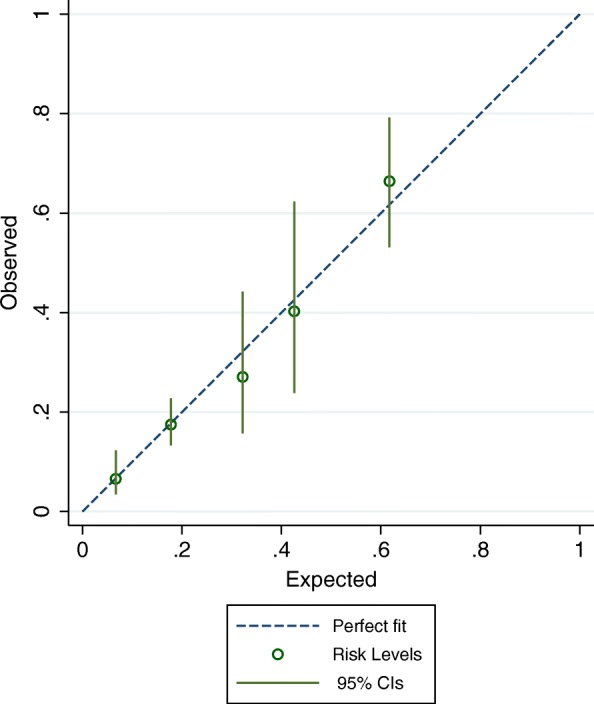
Calibration plot showing the risk of cachexia development after 3 months, as predicted by the risk-level model, plotted against the observed risk

**Fig. 5:**
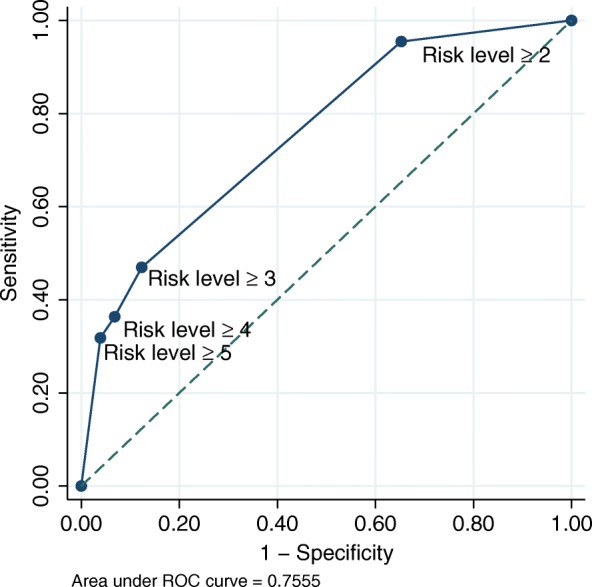
Sensitivity and specificity of cachexia prediction at 3 months when using different cut-offs of risk-level to divide patients into a high or low risk group of cachexia development. Risk-level ≥ 2 yields a high sensitivity (95%), while risk-level ≥ 3 yields a high specificity (88%). No single cut-off yields both a high sensitivity and high specificity

The calibration plot shown in Fig. 4 demonstrates that the risk-level model accurately predicts the observed risk of cachexia development at 3 months, however a Harrell’s C-statistic of 0.71 indicates only modest ability to discriminate between patients who will and will not develop cachexia. Figure 5 presents sensitivity and specificity of cachexia predictions at 3 months for all possible cut-offs between risk-levels in the subsample of patients still alive and remaining in the study after 3 months (*n* = 372). A risk-level ≥ 2 yielded a sensitivity of 95% and a specificity of 35% of predicting cachexia development, while a risk-level ≥ 3 yielded a sensitivity of 47% and a specificity of 88%. Hence, there was no single cut-off with both a high sensitivity and high specificity of predicting cachexia. Area under the curve was 0.76, signifying an accuracy in ability to discriminate between patients with and without cachexia of 76% at 3 months, and is comparable to the corresponding Harrell’s c-statistic.

## Discussion

This study shows that initial weight loss, cancer type, appetite loss and COPD are significant and independent predictors of cachexia in patients with incurable cancer. Based on this, we identify five levels of risk of cachexia development.

The cachexia definition is based mainly on weight loss, and thus, initial minor weight loss below the assigned criteria [9] have been considered indicative of pre-cachexia in several studies [10–12]. The present study confirms that there is an increased risk of further weight loss and eventual development of cachexia when minor weight loss is present. However, the present study also identifies several other risk factors that predict cachexia, independently of weight loss. Among these is cancer type, which has been associated with, and assumed to predict cachexia [14]. The findings of the present study confirm this and demonstrate that a classification of cancer type into low risk cancer (breast cancer and hematologic cancers), high risk cancer (pancreatic and gastric tumours) and neutral risk cancer (all other cancers) significantly predicts cachexia development. However, findings from the CART analysis show that when weight loss is 3% or more, cancer type does not add further to the risk of cachexia development. Contrary to cancer *type*, cancer *stage* (localized vs. metastatic) was not shown to predict cachexia significantly, although a trend was noted in the univariable analysis. The study is not suitable to draw conclusions about cancer stage, however, since the majority of the study population (87%) had metastatic cancer.

Appetite loss is central in the cachexia pathophysiology. It is believed that mediators of cachexia affect the hypothalamus in such a way that the central drive to eat weakens [20]. In turn, this might contribute to an accelerated weight loss through lowered dietary intake. However, conscious control of eating may sometimes prevail over appetite loss [21], and the present study therefore examines both appetite loss and food intake as possible predictors of cachexia. Appetite loss is shown to predict cachexia development independently and appears to be especially important in predicting cachexia in patients with little weight loss (< 3%) and low or neutral risk cancer. Food intake did not independently predict cachexia development, however, and reasons for this might be collinearity (correlation coefficient 0.53) with appetite loss and/or inadequate estimation of food intake.

Patients with COPD had an increased risk of developing cachexia. And although a sensitivity analysis showed that this was partly due to collinearity with lung cancer (which was not explicitly adjusted for in the main analysis), there was still a clear trend towards increased risk of cachexia development. This might be because COPD, as many other chronic diseases, sometimes leads to cachexia. A conservative estimate of the prevalence of cachexia in COPD is 5% [22]. COPD might therefore impose an extra risk of cachexia development on patients with cancer. However, in the subsequent CART-analysis, COPD did not significantly discriminate between groups of patients in terms of cachexia risk, indicating that its role as a risk factor is inferior to the other three significant factors. Notably, heart disease, renal disease and arthritis did not predict cachexia development, although also these conditions are associated with cachexia [22].

Measurements of physical performance applied in the present study (the Karnofsky scale and the physical function scale of the EORTC QLQ C15 PAL) did not predict cachexia development, independently. Analysis of collinearity showed a moderate correlation between Karnofsky and physical function (correlation coefficient 0.54), and collinearity can sometimes explain why two variables that otherwise would be significant, both end up non-significant when present together in a multivariable model. However, this did most likely not explain the lack of significant contribution to the model in the present study as the backward selection in the multivariable analysis ensured that the least significant of the two predictors were rejected from the model before the other. Impaired physical performance is partly caused by the progressive loss of muscle mass that accompanies cachexia [23], and is considered a late symptom [9]. This might be a more likely explanation of why markers of physical performance did not predict cachexia.

### Implications for clinical practice and future research

The present study demonstrates that information on cancer type, appetite loss and COPD improves accuracy of cachexia prediction when added to the established cachexia classifier, weight loss. This is especially true in patients with no or minimal weight loss (< 3%), whereas in patients with weight loss between 3 and 5%, development of cachexia is imminent, regardless of other factors. Based on these predictors, patients can be stratified into five different risk-levels of cachexia development. Cachexia development is not likely if in Risk-level 1, and conversely, for patients in Risk-level 3 or greater the risk of cachexia development is high. As such, the risk-levels enable the clinician to select which patients must be followed more closely with respect to cachexia development and ensure early adequate therapeutic intervention. To the researcher, this could improve patient selection in intervention trials aiming at preventing cachexia, by including only patients at risk of developing cachexia.

No *single* cut-off in this five-level risk ladder has both high sensitivity and high specificity of predicting cachexia. Thus, no single criterion was identified that accurately diagnosed pre-cachexia. Future research should attempt to improve prediction of cachexia development, and thereby improve the diagnosis of pre-cachexia. A likely path towards this aim is to examine the role of inflammatory markers in predicting cancer cachexia. Inflammation is a central part of cachexia pathophysiology and considered a driver of cachexia development [2], and it is likely that markers of systemic inflammation would improve accuracy of cancer cachexia prediction. Thus, the addition of inflammatory markers to the predictors identified in the present study is a necessary next step towards diagnosing pre-cachexia.

### Appraisal of study design

The strength of this study is that it examines factors related to cachexia development in a large longitudinal cohort of patients, and thus enables the identification of factors that predict cachexia development and their relative importance. As is common in many studies in palliative care, the number of missing follow-up observations was relatively high. It is likely that a worsening in physical condition is among the reasons for patients dropping out, and this would decrease statistical power and may introduce a bias. To mitigate this effect, Cox proportional hazards method was used to let each patient contribute with his or her time on the study. Furthermore, to increase power of statistical analysis, patients with insufficient data at baseline, but with sufficient data at first follow-up visit were included with the latest visit as baseline. This was considered appropriate due to the open study design, which allowed inclusion of patients at any time point in their disease trajectory. The CART method is a data mining procedure that is simple to understand and gives an intuitive result. As the calibration plot (Fig. 4) shows, the resulting risk-level model fit the observed risk very well. This is expected when evaluating the model on data from which the model was developed, and the CART methodology may be criticised for creating models that are overfitted to the data, and thus reduce the external validity. By only including significant factors from the Cox model, the risk of overfitting is reduced, and, in addition, the resulting CART model seems clinically plausible. No objective measurements of body composition were available when assessing cachexia. Effect on weight change by accumulation of third space fluids or shifts between fat and muscle mass could therefore not be assessed. However, the adapted definition used in this study has previously been validated [10], and it could be argued that this definition is of greater clinical practical value as objective measures of body composition not always are available in the clinical setting. As mentioned above, no markers of systemic inflammation were assessed as possible predictors of cachexia development. Although the EPCCS study allowed for registration of incidental C-reactive protein measurements performed within 3 days before inclusion, too few observations were available to enable statistical inferences.

## Conclusion

The present study identifies important risk factors for development of cachexia and suggests how these should be combined to optimally stratify patients in terms of cachexia risk. Future research should validate these results and evaluate if addition of markers of systemic inflammation can improve accuracy.

## Data Availability

The dataset generated and analyzed in the current study is not publicly available. This is because there were no financial compensations offered to the research collaborators other than free access to the dataset. As analyses on parts of the dataset are still ongoing, it has not been made publicly available, but may be available from the corresponding author on reasonable request.

## Abbreviations

aPG-SGA: abridged Patient-Generated Subjective Global Assessment
BMI: Body Mass Index
CART: Classification and regression tree analysis
COPD: Chronic obstructive pulmonary disease
EAPC: European association for Palliative Care
EORTC-QLQ: European Organisation for Research and Treatment of Cancer Quality of Life Questionnaire
EPCCS: European Palliative Care Cancer Symptom study
HR: Hazard ratio
IQR: Interquartile range
KPS: Karnofsky performance status
PRC: European Palliative Care Research Centre
PROMs: Patient reported outcome measurements
ROC: Receiver operating characteristic
